# Draft Genome Sequence of the Pelagic Photoferrotroph *Chlorobium phaeoferrooxidans*

**DOI:** 10.1128/genomeA.01584-16

**Published:** 2017-03-30

**Authors:** Sean A. Crowe, Aria S. Hahn, Connor Morgan-Lang, Katherine J. Thompson, Rachel L. Simister, Marc Llirós, Martin Hirst, Steven J. Hallam

**Affiliations:** a Department of Microbiology and Immunology, University of British Columbia, Vancouver, British Columbia, Canada; b Department of Earth, Ocean, and Atmospheric Sciences, University of British Columbia, Vancouver, British Columbia, Canada; c Ecosystem Services, Commercialization Platforms, and Entrepreneurship (ECOSCOPE) Training Program, University of British Columbia, Vancouver, British Columbia, Canada; d Graduate Program in Bioinformatics, University of British Columbia, Vancouver, British Columbia, Canada; e Institute des Sciences de la Vie, Université Catholique de Louvain, Louvain-La-Neuve, Belgium; f Centre for High-Throughput Biology, Department of Microbiology & Immunology, University of British Columbia, Vancouver, British Columbia, Canada; g Canada’s Michael Smith Genome Sciences Centre, BC Cancer Agency, Vancouver, British Columbia, Canada; h Peter Wall Institute for Advanced Studies, University of British Columbia, Vancouver, British Columbia, Canada

## Abstract

Here, we report the draft genome sequence of *Chlorobium phaeoferrooxidans*, a photoferrotrophic member of the genus *Chlorobium* in the phylum *Chlorobi*. This genome sequence provides insight into the metabolic capacity that underpins photoferrotrophy within low-light-adapted pelagic *Chlorobi*.

## GENOME ANNOUNCEMENT

Members of the bacterial phylum *Chlorobi* are best known for growth through anoxygenic photosynthesis, oxidizing reduced sulfur to replace electrons lost from the photosystem during fixation of CO_2_ into biomass (1). One previously cultured *Chlorobi* member,* Chlorobium ferrooxidans* strain KoFox, however, grows by oxidizing ferrous iron [Fe(II)] instead of reduced sulfur (2). Over long stretches of Earth’s history during the Precambrian eons, the oceans were anoxic and Fe(II) rich (ferruginous) (3), making photoferrotrophy the most likely mode of primary production (4, 5). Photoferrotrophs are also implicated in the deposition of the world’s largest iron ore deposits, banded iron formations (BIFs), which deposited from ferruginous oceans (6, 7). *C. ferrooxidans* strain KoFox and all other photoferrotrophs in previous culture collections were isolated from benthic habitats, making them imperfect analogues of the pelagic photoferrotrophs that likely deposited BIFs and supported early Earth primary production. *Chlorobium phaeoferrooxidans* was isolated from the water column of a ferruginous subbasin of Lake Kivu (East Africa), bringing the first known pelagic photoferrotroph into culture (8).

The 16S rRNA gene of *C. phaeoferrooxidans* is 99% similar to that of *C. ferrooxidans* strain KoFox (8), but the two organisms have distinct physiological characteristics (8). *C. phaeoferrooxidans* grows in axenia, whereas KoFox grows in coculture with *Geospirillum* sp. strain KoFum. *C. phaeoferrooxidans* is adapted to low light, using bacteriochlorophyll (BChl) *e* as its light-harnessing pigment, rather than the BChl* c* used in KoFox. The genome of *C. phaeoferrooxidans* was sequenced to determine the metabolic capacity of pelagic photoferrotrophic organisms and inform models for photoferrotrophy during the Precambrian eons.

*C. phaeoferrooxidans* was grown and its DNA extracted and purified as previously described (8, 9). Genomic DNA was subjected to Illumina paired-end library construction and sequenced (MiSeq platform, version 3 chemistry) to generate 16,133,652 paired 250-nucleotide (nt) reads. Quality-filtered reads were assembled into 116 contigs, with an *N*_50_ of 44,807 bp, using ABySS 1.3.5 (10), with default settings. The genome was screened for contaminants based on coverage, G+C composition (49.72%), and conserved single-copy genes. Open reading frame (ORF) prediction and annotation were conducted using MetaPathways (11).

The draft genome is 2.573 Mb, with 2,403 predicted ORFs, one 16S rRNA gene, 51 tRNA genes, and two clustered regularly interspaced short palindromic repeat (CRISPR) arrays. It is 99.45% complete based on 272 conserved single-copy genes (12) and only 71% identical to strain KoFox at the nucleotide level. This genome-level diversity is the basis for defining the two organisms as different species. The* C. phaeoferrooxidans* genome encodes an assimilatory sulfur metabolism but not the canonical oxidative sulfur metabolisms found in most other *Chlorobi*. *C. phaeoferrooxidans* has the genomic capacity to fix carbon using the reverse tricarboxylic acid (TCA) cycle, key genes for the type 1 photosynthetic reaction center, and the Fenna-Matthews-Olson complex characteristic of the *Chlorobi* (13). The genome also encodes an outer membrane cytochrome (cyc2_PV-1_) implicated in Fe(II) oxidation in the microaeorphillic Fe(II)-oxidizing zetaproteobacterium *Mariprofundus ferrooxidans* strain PV1 (14). These data expand the metabolic blueprint for photoferrotrophy in the *Chlorobi* and provide an opportunity to define the physiological basis for BIF deposition and primary production in the Precambrian oceans.

### Accession number(s).

This whole-genome shotgun project has been deposited in GenBank under the accession no. MPJE00000000. The version described in this paper is the first version.
